# Gene Expression Differences Predict Treatment Outcome of Merkel Cell Carcinoma Patients

**DOI:** 10.1155/2014/596459

**Published:** 2014-01-30

**Authors:** Loren Masterson, Bryan J. Thibodeau, Laura E. Fortier, Timothy J. Geddes, Barbara L. Pruetz, Rajwant Malhotra, Richard Keidan, George D. Wilson

**Affiliations:** ^1^Department of General Surgery, Beaumont Health System, Royal Oak, MI 48073, USA; ^2^Department of Beaumont BioBank, Beaumont Health System, 3811 West 13 Mile Road 105-RI, Royal Oak, MI 48073, USA; ^3^Department of Anatomic Pathology, Beaumont Health System, Royal Oak, MI 48073, USA

## Abstract

Due to the rarity of Merkel cell carcinoma (MCC), prospective clinical trials have not been practical. This study aimed to identify biomarkers with prognostic significance. While sixty-two patients were identified who were treated for MCC at our institution, only seventeen patients had adequate formalin-fixed paraffin-embedded archival tissue and followup to be included in the study. Patients were stratified into good, moderate, or poor prognosis. Laser capture microdissection was used to isolate tumor cells for subsequent RNA isolation and gene expression analysis with Affymetrix GeneChip Human Exon 1.0 ST arrays. Among the 191 genes demonstrating significant differential expression between prognostic groups, keratin 20 and neurofilament protein have previously been identified in studies of MCC and were significantly upregulated in tumors from patients with a poor prognosis. Immunohistochemistry further established that keratin 20 was overexpressed in the poor prognosis tumors. In addition, novel genes of interest such as phospholipase A2 group X, kinesin family member 3A, tumor protein D52, mucin 1, and *KIT* were upregulated in specimens from patients with poor prognosis. Our pilot study identified several gene expression differences which could be used in the future as prognostic biomarkers in MCC patients.

## 1. Introduction

Merkel cell carcinoma (MCC) was first described in 1972 by Toker, which was initially thought to be derived from sweat glands [1]. In 1978, dense-core granules were found in these tumors consistent with neuroendocrine cells, and in particular Merkel cells [2]. MCC has gained recent attention not only due to its association with newly discovered Merkel polyomavirus but also due its increase in incidence [3]. In fact, the age-related incidence of MCC has nearly tripled from 1986 to 2001 [3]. Risk factors include advanced age, ultraviolet light exposure, and chronic immunosuppression. MCC are most commonly located in the head and neck, often accompanied by other primary cutaneous neoplasms, consistent with ultraviolet light exposure.

Prognosis of MCC is poor. Patients presenting with distant disease have two-year survival of only 32% [4]. Only 50% of patients have localized disease (Stage 1) at time of diagnosis. Staging is primarily based on tumor size and the extent of disease at the time of diagnosis [5]. Those patients diagnosed with disease and tumor size <2 cm without nodal involvement have a ten-year mortality between 29 and 39% [6]. This is more than four times the mortality rate for patients with melanoma diagnosis with the same disease stage [7]. Currently, prognosis is best determined by the extent of disease of initial diagnosis.

Due to rarity of MCC, prospective clinical trials have proven unfeasible [8]. More recently, several studies have focused on biomarker analysis in order to identify prognostic factors [9–11]. In this study, we performed global gene expression analysis of tumor specimens from patients with good, poor, and moderate outcomes to identify novel genes and pathways that might identify new prognostic markers or new treatment targets.

## 2. Materials and Methods

### 2.1. Patients and Specimens

We interrogated the Beaumont Hospital tumor registry for all patients treated for MCC within the last 10 years; 62 patients were identified. Patients were excluded who did not have tumor resection or tissue diagnosis at our institution due to lack of access to specimen. Patients were also excluded with inadequate followup or if their specimens failed to pass quality control measures for gene expression studies. All tissue paraffin block specimens were retrieved from anatomic pathology for validation and identification of disease by a single dermatopathologist (RM). After verification of tissue, MCC patients were stratified into one of three groups based upon status at 24 months following diagnosis. Poor prognosis patients either presented with or progressed to distant metastasis. Patients were considered to have a moderate prognosis if they presented with recurrent local disease or nodal disease at presentation but had no progression during followup of fewer than 24 months. Patients with favorable prognosis had local disease with no nodal disease at presentation and no progression during followup of at least 24 months [12]. Seventeen MCC patients satisfied all the inclusion criteria (6 poor prognosis, 3 moderate prognosis, and 8 good prognosis patients).

### 2.2. Laser Capture Microdissection (LCM)

For LCM, 5 *μ*m sections were cut from formalin-fixed paraffin-embedded (FFPE) tissue and mounted onto polyethylene naphthalate membrane glass slides (two sections per slide). Prior to deparaffinization, slides were placed into an oven at 60°C for 25 minutes then stained and dehydrated through a series of graded ethanol and xylene steps. Within 3 hours of sectioning, the pathologist-identified tumor areas were microdissected with both UV and IR lasers, where appropriate, using the Arcturus^XT^ Microdissection System (Molecular Devices) onto CapSure HS LCM Caps (Molecular Devices). Four caps were used per graded region from the dissection of 1–4 sections representing approximately 5,000 tumor cells (1-2 mm^2^ area) captured per cap.

### 2.3. RNA Isolation

Total RNA was isolated from the LCM tissues using the PureLink FFPE RNA Isolation Kit (Invitrogen). The transfer film with the attached dissected material was separated from each CapSure HS LCM Cap and placed in deparaffinization melting buffer at 72°C for 10 min and then treated with Proteinase K at 60°C for 45 min. Following the manufacturer's protocol, total RNA was purified using spin cartridge technology, treated with DNase, quantified (Nanodrop 8000, Thermo Scientific), and stored at −80°C. RNA integrity was determined just prior to processing for expression microarray analysis. RNA integrity numbers ranged from 2.0 to 2.4, as determined by Bioanalyzer analysis (Agilent).

### 2.4. Whole Transcriptome (WT) Amplification, Purification, Fragmentation, and Hybridization

Total RNA was amplified and labeled using the WT-Ovation FFPE System V2, WT-Ovation Exon Module, and the Encore Biotin Module (NuGEN, Inc. San Carlos, CA, USA), which enables amplification and target preparation of the low quality RNA from FFPE, LCM samples. Amplifications were performed with 100 ng total RNA input following procedures described in the WT-Ovation FFPE System user guide. Target preparation for Affymetrix Human Exon 1.0 ST Arrays (Affymetrix, Santa Clara, CA, USA) was performed using 5 *μ*g amplified cDNA and the Encore Biotin Module following the manufacturer's recommendations. Hybridization, washing and staining (with GeneChip Fluidics Station 450, Affymetrix), and scanning (with GeneChip Scanner 3000) were performed following manufacturer's protocols. Array scans from this process yielded signal intensities comparable to arrays prepared from high quality RNA from cell lines (data not shown). This method has also been independently reported recently to provide optimal RNA hybridization [13].

### 2.5. Gene Expression Analysis

The CEL files containing the raw intensity data from the Affymetrix GeneChip arrays were imported into Partek Genomics Suite (version 6.6 beta, build 6.11.1115) and normalized using the robust multichip average with a guanine-cytosine content background correction, quantile normalization, log2-transformation, and median polish probeset summarization. Exons were then summarized to genes using the average of the probesets. Differentially expressed genes were identified using ANOVA with two factors: prognosis and scan date (random variable). Unsupervised hierarchical clustering was carried out using Partek software (Partek Inc., St. Louis, MO, USA). Hierarchical clustering analysis was performed using Euclidean distance as similarity measure and average linkage for the agglomerative method. Gene set enrichment analysis (GSEA) and pathway analysis were performed using Pathway Studio 9.0 (Ariadne Genomics, Rockville, MD, USA). The data discussed in this publication have been deposited in NCBI's Gene Expression Omnibus [14] and are accessible through GEO Series accession number GSE36150.

### 2.6. Immunohistochemistry

Tissue sections from the corresponding FFPE tissue blocks were placed on slides, underwent high pH antigen retrieval, and were subsequently incubated with one of the following primary antibodies: mouse monoclonal anti-human keratin 20 (prediluted, Dako IS777), mouse monoclonal anti-human mucin 1 (1 : 100, Leica, NCL-HGM-45 mL), and rabbit polyclonal anti-human KIT (1 : 250, Dako A4502), rabbit polyclonal anti-human KIF3A (1 : 1200, Sigma K3513). The slides were then linked and visualized using the Dako Envision Flex system on a Dako Autostainer. Immunohistochemistry was scored based on intensity of staining with a 4-tier system (0 = absent, 1 = weak, 2 = intermediate, and 3 = intense). To address interobserver variability, scoring was performed by 2 independent investigators, and scores were averaged. Fisher's exact probability test was conducted comparing good and poor prognosis cohorts [15].

## 3. Results

### 3.1. Identification and Isolation of Merkel Cell Samples

The patient demographics and lesion characteristics are outlined in Table 1. In this small cohort of patients, there was almost even number of male and females (53% versus 47%), and 53% (9 of 17 patients) of lesions were located on the extremities. While the majority (59%, 10 of 17) of patients were Stage II, 75% (3 of 4) of patients diagnosed with Stage III tumors had a poor prognosis.

**Figure 1:**
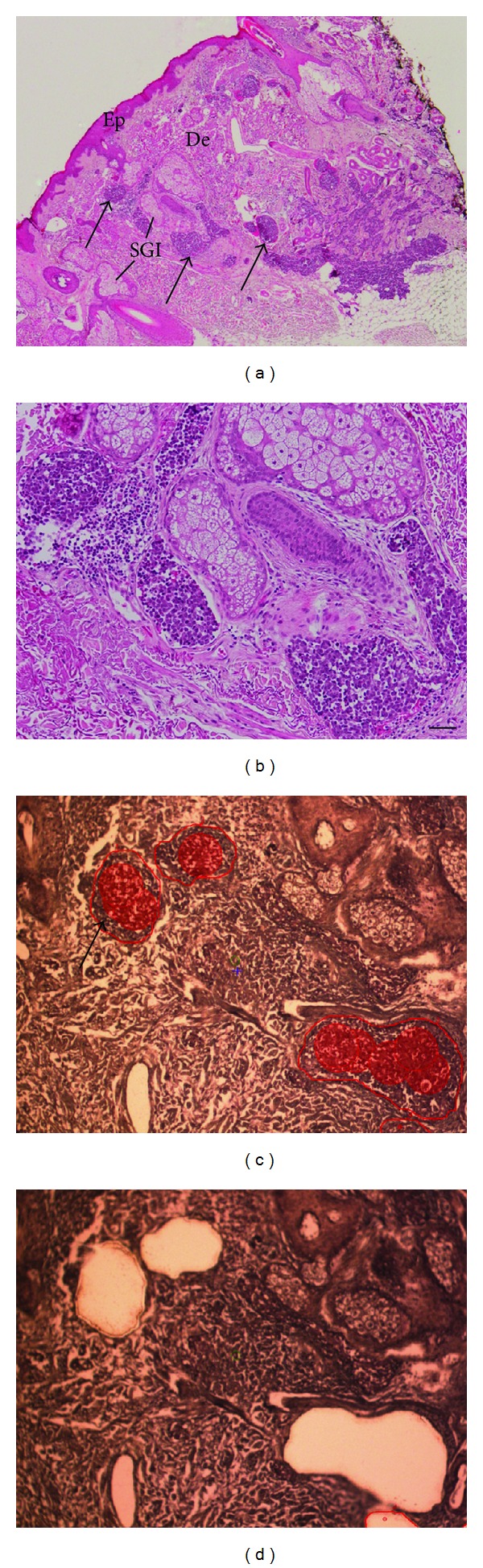
Representative example of histology of Merkel cell carcinoma from a tumor with poor prognosis. (a) Low power hematoxylin and eosin (H&E) stained photomicrograph. Arrows indicate regions of MCC to be microdissected. Dermis (De), epidermis (Ep), and sebaceous gland (SGI) are also indicated. (b) High power H&E image of same MCC. (c) Arcturus glass membrane hematoxylin counterstained serial section from same MCC. Areas to be laser capture microdissected are indicated by the arrow and red shading. The red punctate shading marks subsequent laser positioning. Using the Arcturus^XT^ Microdissection System (molecular devices), these regions were transferred onto CapSure HS LCM Caps (Molecular Devices). Four caps were used per patient sample from the dissection of 1–4 sections representing approximately 5,000 tumor cells (1-2 mm^2^ area) captured per cap. (d) Image of the same membrane cut section after LCM. Magnification: (a) at 2X; (b), (c), and (d) at 10X. Scale bar = 50 *μ*m.

Laser capture microdissection was used to isolate and remove as pure a population of tumor cells as possible based on morphological identification by a dermatopathologist.  Figure 1 shows a typical lesion from a 49-year-old female with a Stage 2 lesion on a lower extremity; this lesion was from a poor prognosis patient that required surgery and salvage chemotherapy. The lesion showed a nodular arrangement of cells within the dermis with sparse cytoplasm with uniform, monotonous medium-sized nuclei (Figures 1(a) and 1(b)). The histological composition of the tumor clearly shows the importance of the laser capture microdissection approach taken in this study due to the presence of dermal and adnexal structures. Figures 1(c) and 1(d) show the selected outlined areas for dissection before and after laser capture microdissection.

### 3.2. Gene Expression Changes in MCC Related to Prognosis

The 17 samples of MCC were analyzed using the Human Exon 1.0 ST array from Affymetrix. Two arrays (one good prognosis and one poor prognosis) failed to pass array quality control metrics. In a comparison of the patients with poor prognosis (*n* = 7) versus those with good prognosis (*n* = 5), 191 genes were found to be differentially expressed (*P* ≤ 0.05 and 1.5-fold cutoff, Table S1 in the Supplementary Material available online at http://dx.doi.org/10.1155/2014/596459). This includes 127 genes that are overexpressed in tumors from poor prognosis patients and 64 genes that are underexpressed. *NEFM* (neurofilament, medium polypeptide), *PLA2G10* (phospholipase A2, group X), and *KRT20* (keratin 20) are upregulated more than 3-fold in tumors with poor prognosis. *CYP2A6* (cytochrome P450, family 2, subfamily A, polypeptide 6), *MCART1* (mitochondrial carrier triple repeat 1), and *KRTAP19-5* (keratin associated protein 19-5) were downregulated at least 2.8-fold in poor prognosis samples.

**Figure 2:**
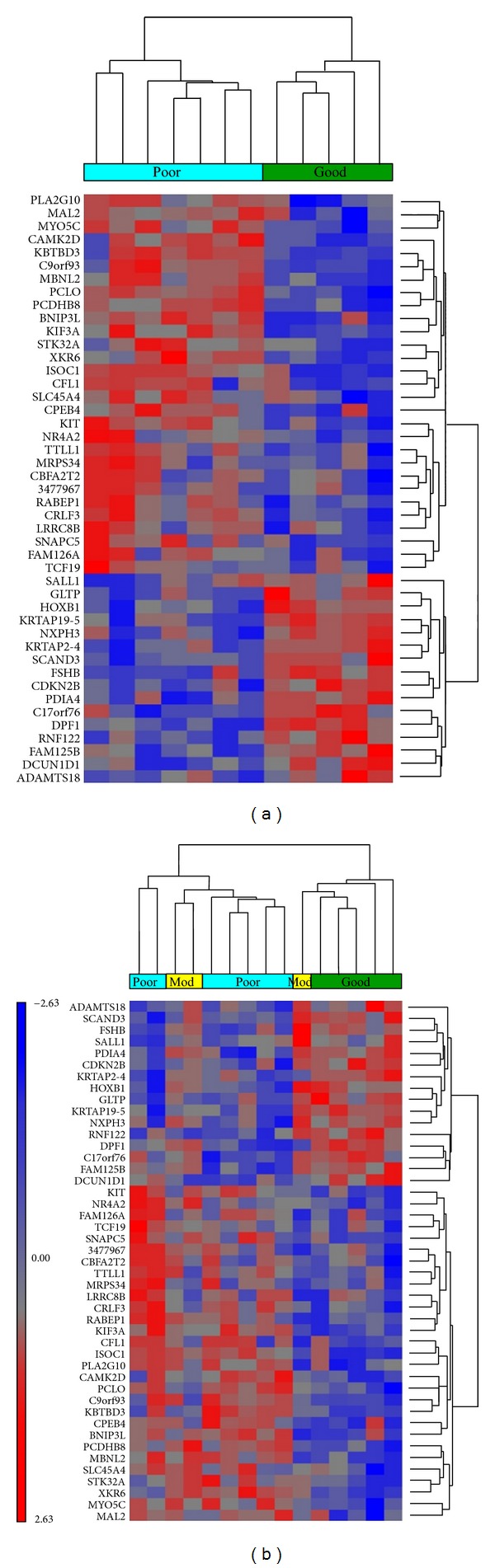
Hierarchical clustering of Merkel cell carcinoma samples. Samples were clustered based upon the expression pattern of 45 differentially expressed genes between tumors of poor and good prognosis patients (*P* ≤ 0.01 and 1.5-fold cutoff). Individual samples are represented on the *x*-axis with poor prognosis patients in light blue (“poor”), good prognosis in green (“good”), and moderate prognosis in yellow (“mod”). Differentially expressed genes are shown on the *y*-axis. Clustering of (a) good and poor prognosis patients; (b) good, moderate, and poor prognosis patients. The scale of gene expression values are standardized such that genes are shifted to a mean of zero and scaled to a standard deviation of one.

Among the 191 differentially expressed genes, there is a subset of 45 genes that meet the stricter conditions of *P* ≤ 0.01. Hierarchical clustering of the MCC samples was performed based upon these 45 differentially expressed genes. Clustering based upon the expression of these genes robustly delineated between the good and poor prognosis patients (Figure 2(a)). Inclusion of the 3 samples from patients with a moderate prognosis resulted in 2 patients clustering with the poor prognosis patients and 1 with the good prognosis patients (Figure 2(b)). The annotation for AJCC (American Joint Committee on Cancer) stage in these figures indicates that clustering based upon prognosis dependent gene expression is independent of stage.

### 3.3. Subnetwork Analysis

**Figure 3:**
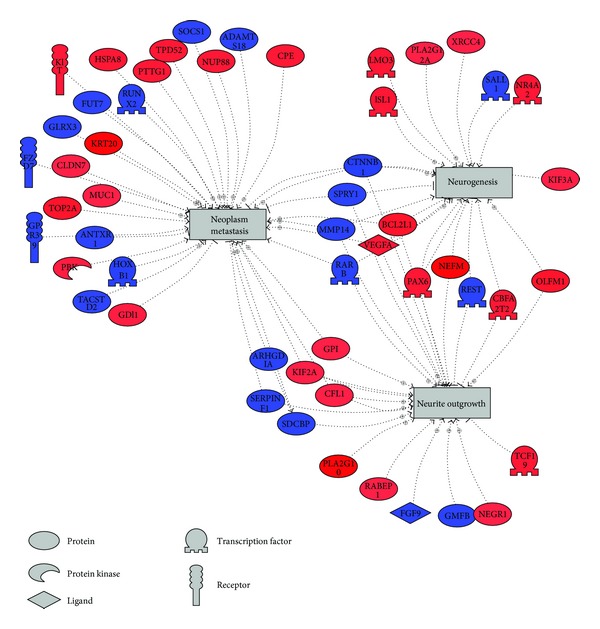
Subnetwork of genes involved in regulating neoplasm metastasis, neurite outgrowth, and neurogenesis. Genes in red are upregulated in poor prognosis; blue, downregulated.

In order to better understand the biological context of the gene expression differences between MCC from good and poor prognosis patients, the expression microarray data was analyzed using Ariadne Pathway Studio's Subnetwork Enrichment Analysis tool [16, 17]. Pathway Studio utilizes MedScan, the literature mining program that searches publicly the available literature such as PubMed for relationships between entities [18]. A subnetwork consists of a single seed (i.e., disease or cell process) and genes associated to this seed by regulation of/by the seed [19]. The expression microarray dataset is interrogated with no prior significance filtering, and enrichment of the subnetwork is determined by both the level of regulation in the network and the size of the network. One type of network consists of genes associated with regulating diseases; that is, the “seed” of the subnetwork is a disease or disease condition. Interestingly, the top subnetwork identified is “genes associated with regulating neoplasm metastasis” (Figure 3). The visualized subnetwork was limited to include only those genes that were differentially expressed at ANOVA *P* ≤ 0.10 and a 1.5-fold cutoff. Less stringent conditions have previously been shown to be more appropriate for pathway analysis [20, 21].

Subnetwork enrichment analysis was then utilized to discover cell processes that are highly regulated by gene expression differences between tumors of good and poor prognosis patients (Table 2). Fourteen of the top twenty categories are related to growth, including regeneration, mitosis, invasive growth, and cell motility. In addition, two categories (neurite outgrowth and neurogenesis, Figure 3) suggest a connection to the neuroendocrine nature of MCC.

### 3.4. Immunohistochemistry

**Figure 4:**
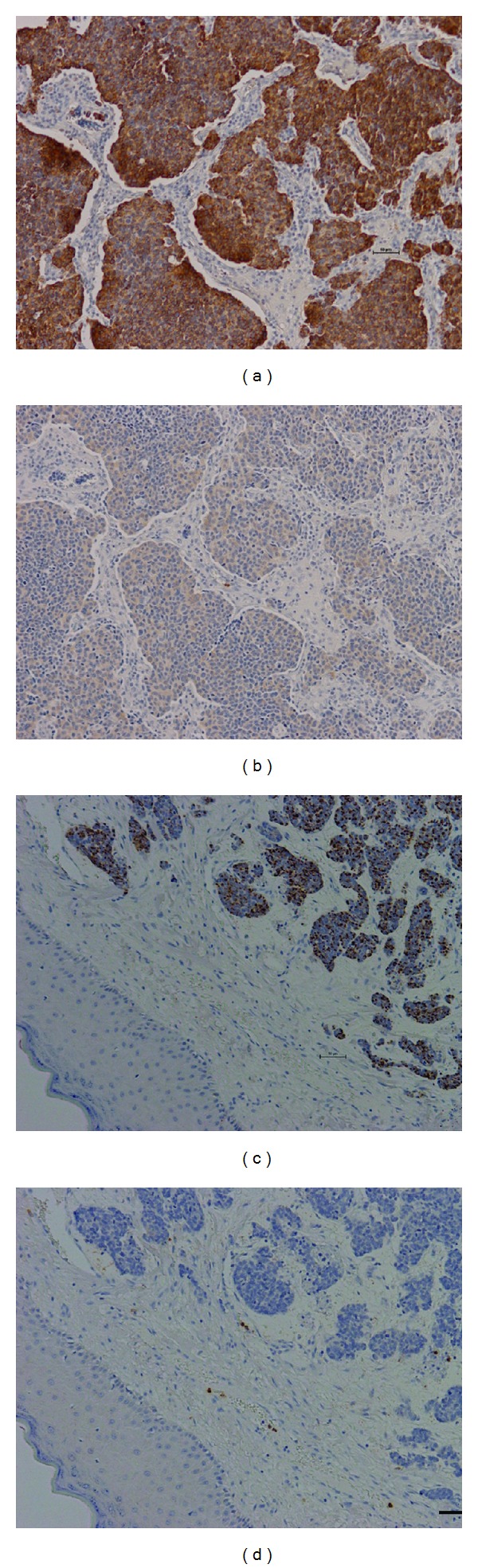
Immunohistochemistry in representative Merkel cell carcinoma tumors with good and poor prognoses. (a) intermediate KRT20 staining in poor prognosis tumor. (b) Weak KIF3A staining in poor prognosis tumor. (c) Weak punctate KRT20 staining in good prognosis tumor. (d) Negative (absent) KIF3A staining in good prognosis tumor. All images were taken at 10X magnification with Nikon digital DS-Fi1 high definition color camera on the Nikon Eclipse 90i. Scale bar = 50 *μ*m.

Several differentially expressed genes were further explored at the protein level. Immunohistochemistry was utilized to examine the protein products of MUC1, KIT, KIF3A, and KRT20 (Table 3). While the majority of all MCC samples (88%, 15 of 17) were positive for KRT20, the poor prognosis samples (Figure 4(a)) showed stronger staining than those from good prognosis patients (Figure 4(b)). Using Fisher's exact probability test comparing absent/weak staining to intermediate/intense staining, this result was significantly significant in this pilot study (*P* ≤ 0.03). This agrees with the overexpression seen in the gene level analysis. KIF3A protein was detectable in 1 of 6 poor prognosis patient samples (Figure 4(c)), but no protein was evident in samples from the moderate or good prognosis patients (Figure 4(d)). MUC1 protein was undetectable in almost all samples with the exception of low level staining in the sample from one good prognosis patient. The protein levels of KIT did not corroborate the gene expression results. Despite 83% of good prognosis (5 of 6) and 75% of poor prognosis (6 of 8) samples being KIT positive, there was no pattern to the level of staining based upon prognosis (*P* ≤ 0.54).

## 4. Discussion

Diagnosis of Merkel cell carcinoma can be difficult. Current treatment consists of surgical removal with very wide excision borders. Virtually, all patients receive radiation following wide surgical excision, while patients with positive lymph nodes or metastatic disease at time of diagnosis may be candidates for chemotherapy. No standard chemotherapy protocol has yet been established for the treatment of MCC. Because of the morphological and immunohistochemical similarity of MCC to small cell lung carcinoma (SCLC), chemotherapy has been performed with protocols based largely on agents active in SCLC. A wide variety of chemotherapeutic agents have been discussed, including cytostatic drugs such as cyclophosphamide, doxorubicin, epirubicin, vincristine, etoposide, cisplatin, carboplatin, 5-fluorouracil, dacarbazine, mitoxantrone, bleomycin, and iphosphamide. Unfortunately, reports to date consist of only small studies and anecdotal evidence [22–24]. There is a need to move away from the largely empirical clinical management of MCC and find ways to understand and individualize treatment.

The ultimate goal of the study was to find markers that could identify poor prognosis patients at the time of diagnosis; these patients could benefit from increased or altered postsurgical therapy. In order to discover these novel biomarkers, expression microarray technology was utilized to examine global gene expression differences between patients that demonstrated a known prognosis (good prognosis or poor prognosis) based upon status 24 months following resection. This analysis resulted in a set of 191 genes being identified as differentially expressed. Hierarchical clustering of the patient samples based upon the expression of a subset of these genes (Figure 2(a)) demonstrated their utility in discriminating between the two prognoses. While definitive conclusions cannot be drawn due to the limited numbers in this pilot study, perhaps more interesting is the result of adding the patients with moderate prognosis to the clustering (Figure 2(b)). The good and poor prognosis patients remain segregated into two main clusters while one moderate patient cluster with the good prognosis patients and two with the poor prognosis patients. The patient that clustered with the good prognosis patients was a 71-year-old female whose primary was located on the breast. The patient samples that clustered with the poor prognosis patient samples were from an 84-year-old male with the primary located on the arm and an 81-year-old male with the primary on the lip. The moderate prognosis patients that group with the poor prognosis group represented the more “typical” MCC patient: male, older, and with the primary on a sun-exposed area. The moderate patient that clusters with the good prognosis patients had the primary at an atypical location and was female. In general, female MCC patients have been shown to have a better prognosis than male patients [6]. Longer term followup of these three patients could help to determine the accuracy of these projections to the good or poor prognosis groups based upon the expression of these 45 differentially expressed genes.

Further characterization of the expression microarray data using subnetwork enrichment analysis revealed that gene expression associated with neoplasm metastasis and neurite outgrowth was highly regulated between samples of patients with a good and poor prognoses. The neoplasm metastasis associated genes *KRT20*, tumor protein D52 (*TPD52*), *MUC1*, and *KIT* are upregulated in the poor prognosis tumors while homeobox B1 (*HOXB1*) is downregulated. *TPD52*, upregulated nearly 2-fold in tumors of poor prognosis patients, has been shown to be overexpressed in colorectal cancer [25] and ovarian cancer [26], and genomic amplification of *TPD52* has been seen in prostate cancer [27] and breast cancer [28]. *KIT* is also upregulated in the poor prognosis specimens. KIT is a transmembrane receptor for mast cell growth factor, also known as stem cell factor. Studies with MCC cell lines show that KIT is activated by paracrine or autocrine tumor cell-derived SCF which stimulates growth of MCC in vitro [29]. In one study looking at KIT expression in MCC tumors, Andea et al. showed dramatic survival differences between patients based upon KIT protein expression with high levels of KIT resulting in decreased 5-year survival [30]. *HOXB1*, which is downregulated in poor prognosis MCC, is a highly conserved transcription factor that plays an important role in morphogenesis and has been shown to be involved the metastatic potential of pancreatic cancer [31].

Genes associated with neurite outgrowth include the genes for *NEFM* and *PLA2G10*. Both of these genes are upregulated more than 3.5-fold in tumors of poor prognosis patients. NEFM encodes a type IV intermediate filament medium chain and is commonly used as a biomarker of neuronal damage. In one study, neuroendocrine cell lines expressed *NEFM* while nonneuroendocrine cell lines did not [32]. Along with KRT20, NEFM was shown to be effective at discriminating MCC from small cell lung carcinoma with the majority of MCC being positive for both NEFM and KRT20 while NEFM and KRT20 were almost completely absent from small cell lung carcinoma [33].

In addition to these genes, there were other genes that were of interest and associated with cancer development or progression including dimethylarginine dimethylaminohydrolase 1 (*DDAH1*), kinesin family member 3A (*KIF3A*), and *MUC1* which are all upregulated in the poor prognosis samples. The enzyme encoded by *DDAH1* is involved in nitric oxide generation through regulation of methylarginines. Nitric oxide metabolism has been implicated in carcinogenesis, tumor progression, angiogenesis, and response to therapy [34]; in particular, overexpression of *DDAH1* resulted in increased tumor growth and vascularization in a model of glioma tumorigenesis [35]. KIF3A activity is linked to Hedgehog signaling, which is upregulated in MCC [36]. KIF3A is required for the development of cilia which are present on most cells and are implicated in transducing Hedgehog signals during development [37]. The MUC1 protein is expressed on the apical surface of epithelial cells that line the mucosal surfaces of many different tissues. In a study by Kurzen et al., MUC1 was found to be expressed in Merkel cells and in about 82% of all MCC and 66% of metastases [38].

When examining the protein products of several of these differentially expressed genes by immunohistochemistry, the protein and gene expression results were in agreement for only 1 of 4 genes. KRT20 demonstrated increased mRNA and protein expression in the tumors of poor prognosis patients. KRT20 is an intermediate filament protein responsible for the structural integrity of epithelial cells. Our expression microarray analysis indicated 3.0-fold overexpression in poor prognosis tumors while the immunohistochemistry results confirmed an increase in the protein level. In earlier studies, 88–100% of MCC was positive for KRT20 [33, 39]. In particular, KRT20 was used as a marker for differentiating MCC from small cell carcinoma of the lung [33, 40]. Micrometastatic foci in sentinel lymph node biopsies stained strongly for KRT20 [41]. Unlike the positive results with KRT20, protein levels of KIF3A and MUC1 did not display a positive association with gene expression results. Despite the expression microarray evidence indicating robust gene expression in both prognoses groups, KIF3A and MUC1 proteins were each detected in only 1 sample. Similarly, the KIT protein levels did not correspond to prognosis group despite 67% (10 of 15) of MCC samples staining positive for KIT. This did, however, validate a previous study that found that 65% of MCC were KIT positive [42]. While there is limited agreement between the gene and protein expression data, there is substantial evidence in the literature to indicate that there is significant regulation of protein levels subsequent to mRNA transcription, thus allowing for differing results when comparing gene expression with protein expression [43].

The results of this study further develop a recently published gene expression analysis by Harms et al. [44]. While our comparison was among MCC patients based upon prognosis, they compared among MCC positive or negative for Merkel cell polyomavirus, squamous cell carcinoma (SCC), and normal skin. Intriguingly, the results comparing MCC with SCC and normal skin resulted in similar results reported here. Piccolo (*PCLO*), *KRT20*, and *KIT* were upregulated in MCC compared to SCC just as these 3 genes were upregulated in poor prognosis patient samples compared to good prognosis patient samples. In addition, functional classes found to be altered between MCC and SCC included categories such as nervous system development, neuron development, and neuron projection morphogenesis which is similar to those found in Table 2. In the comparison of MCC to normal skin, NEFM was found to be highly upregulated in MCC while our results showed that NEFM was upregulated more than 4-fold in poor prognosis patient samples.

## 5. Conclusions

In recent years, research has made rapid progress in identifying biological features of Merkel cell carcinoma; nevertheless, MCC represents a considerable challenge due to its relative rarity. While multiple studies have identified several biomarkers, prognostic value of these biomarkers has yet to be determined. This study is among the first to use LCM-captured MCC cells to investigate specific gene expression changes associated with prognosis of the tumor. Several of the gene expression changes presented here further implicate their role in MCC. Gene expression of *KRT20*, *KIT*, *MUC1*, and *NEFM* has been identified in earlier MCC studies. In addition, several new potential biomarkers were identified. Genes such as *TPD52*, *HOXB1*, and *KIF3A* have been implicated in other cancer types and are connected with MCC-associated signaling. Furthermore, KRT20 demonstrated elevated protein levels in poor prognosis patient samples. This complements results from Harms et al. showing elevated KRT20 protein in MCC compared with SCC. From these results, we conclude that genes such as *KIT*, *HOXB1*, and *KIF3A* may provide valuable insights into the biology of MCC and the possibility remains that novel therapeutic modalities may be discovered with continued understanding of these biomarkers. Additionally, protein expression levels of KRT20 will be of importance in evaluating the prognosis of MCC. Given the limited sample numbers in this pilot study, we aim to expand this study through collaborations with other institutions. Ongoing studies, including further analysis of data with longer patient followup and an assessment of the influence of polyomavirus in these patients, will be necessary in order to elucidate the ultimate potential of these new biomarkers.

## Supplementary Material

Table S1: lists the 191 genes that are differentially expressed between patients with good prognosis and those with poor prognosis (p ≤ 0.05 and 1.5-fold cutoff). Genes selected for follow-up protein analysis by immunohistochemistry are noted.

## Figures and Tables

**Table 1:** Clinical characteristics of the 17 MCC patients utilized in study.

Feature	Good prognosis	Moderate prognosis	Poor prognosis	Total
	*n* = 6 (35%)	*n* = 3 (18%)	*n* = 8 (47%)	*n* = 17
Gender				
Male	3 (50%)	2 (67%)	4 (50%)	9 (53%)
Female	3 (50%)	1 (33%)	4 (50%)	8 (47%)
Age, years: median, range	71.7 (54–82)	78.7 (71–84)	68.8 (44–83)	
Location				
Head and neck	3 (50%)	1 (33%)	2 (25%)	6 (35%)
Upper extremity	2 (33%)	1 (33%)	2 (25%)	5 (30%)
Lower extremity	1 (17%)	0 (0%)	3 (37.5%)	4 (23%)
Torso	0 (0%)	1 (33%)	1 (12.5%)	2 (12%)
AJCC stage at diagnosis				
(I)	2 (33%)	1 (33%)	0 (0%)	3 (18%)
(II)	3 (50%)	2 (67%)	5 (62.5%)	10 (59%)
(III)	1 (17%)	0 (0%)	3 (37.5%)	4 (23%)

**Table 2:** Subnetworks of genes involved in regulating cell processes that are highly regulated between patients with poor and good prognoses.

Gene set seed	*P* value
Neurite outgrowth	3.36*E* − 06
Regeneration	3.87*E* − 05
Kinetochore assembly	8.02*E* − 04
Hair cell differentiation	9.68*E* − 04
Olfactory bulb development	9.79*E* − 04
Organogenesis	9.96*E* − 04
Sertoli cell proliferation	1.10*E* − 03
Spindle assembly	1.72*E* − 03
Neural crest cell development	2.18*E* − 03
Chondrogenesis	2.41*E* − 03
Ureteric bud branching	2.76*E* − 03
Protein polyubiquitination	2.79*E* − 03
Ion channel clustering	2.85*E* − 03
Chondrocyte differentiation	2.86*E* − 03
Cytokinesis	2.86*E* − 03

**Table 3:** Immunohistochemistry results for KIF3A, KIT, KRT20, and MUC1. Results were grouped into absent/weak and intermediate/intense staining. Fisher's exact probability test compared good and poor prognosis results.

	Good prognosis	Moderate prognosis	Poor prognosis	*P* value
KIF3A				
Absent/weak	6	3	8	—
Intermediate/intense	0	0	0	
KIT				
Absent/weak	4	2	7	*P* ≤ 0.54
Intermediate/intense	2	1	1	
KRT20				
Absent/weak	6	1	3	*P* < 0.03
Intermediate/intense	0	2	5	
MUC1				
Absent/weak	6	3	8	—
Intermediate/intense	0	0	0	

(—: *P* value not calculated due to *n* = 0 in intermediate/intense class).
