# Supplementary material for: Gene Expression Differences Predict Treatment Outcome of Merkel Cell Carcinoma Patients
Source: J Skin Cancer. 2014 Jan 30;2014:596459. doi: 10.1155/2014/596459 (PMC3929072; doi:10.1155/2014/596459)
Supplement: Supplementary file 1 — Table S1: lists the 191 genes that are differentially expressed between patients with good prognosis and those with poor prognosis (p ≤ 0.05 and 1.5-fold cutoff). Genes selected for follow-up protein analysis by immunohistochemistry are noted. [file 596459.f1.pdf]

Supplementary Table 1. Genes that are differentially expressed in the poor prognosis patients compared to the good prognosis patients (p≤0.05 and 1.5-fold cutoff). * indicates p≤0.01; †, subsequent immunohistochemistry follow-up.

RefSeq	Gene Symbol	Gene Name	Fold Change (Poor vs Good)
NM_005382	NEFM	neurofilament, medium polypeptide	4.32
NM_003561	*PLA2G10	phospholipase A2, group X	3.55
NM_019010	†KRT20	keratin 20	3.05
NM_144778	*MBNL2	muscleblind-like 2 (Drosophila)	2.51
NM_003196	TCEA3	transcription elongation factor A (SII), 3	2.35
NM_001130688	HMGB2	high-mobility group box 2	2.25
NM_005089	ZRSR2	zinc finger (CCCH type), RNA-binding motif and serine	2.23
NM_001100117	RIMS2	regulating synaptic membrane exocytosis 2	2.15
NM_052886	*MAL2	mal, T-cell differentiation protein 2	2.15
NM_001008388	CISD2	CDGSH iron sulfur domain 2	2.11
NR_026666	*MGC16384	hypothetical LOC114130	2.11
NM_003539	HIST1H4D	histone cluster 1, H4d	2.04
NM_012137	DDAH1	dimethylarginine dimethylaminohydrolase 1	2.03
NM_001025252	TPD52	tumor protein D52	1.99
NM_007054	*†KIF3A	kinesin family member 3A	1.99
NM_001080431	*SLC45A4	solute carrier family 45, member 4	1.98
NM_032119	GPR98	G protein-coupled receptor 98	1.98
NM_002456	†MUC1	mucin 1, cell surface associated	1.96
NM_014711	CP110	CP110 protein	1.95
NM_014510	*PCLO	piccolo (presynaptic cytomatrix protein)	1.94
NM_017681	NUP62CL	nucleoporin 62kDa C-terminal like	1.93
NM_030627	*CPEB4	cytoplasmic polyadenylation element binding protein 4	1.93
NM_006049	*SNAPC5	small nuclear RNA activating complex, polypeptide 5	1.93
NM_018365	MNS1	meiosis-specific nuclear structural 1	1.92
NM_080676	MACROD2	MACRO domain containing 2	1.91
NM_005093	*CBFA2T2	core-binding factor, runt domain, alpha subunit 2; translocated to, 2	1.91
NM_005988	SPRR2A	small proline-rich protein 2A	1.90
NM_023037	FRY	furry homolog (Drosophila)	1.89
NM_001134476	*LRRC8B	leucine rich repeat containing 8 family, member B	1.89
AB073660	*XKR6	XK, Kell blood group complex subunit-related family, member 6	1.88
BC058160	RPL36AP40	ribosomal protein L36a pseudogene 40	1.86
NM_001873	CPE	carboxypeptidase E	1.86
NM_014402	UQCRQ	ubiquinol-cytochrome c reductase, complex III subunit VII	1.85

NM_000222	*†KIT	v-kit Hardy-Zuckerman 4 feline sarcoma viral oncogene homolog	1.84
NM_003538	HIST1H4A	histone cluster 1, H4a	1.84
NM_017785	CCDC99	coiled-coil domain containing 99	1.83
NM_015879	ST8SIA3	ST8 alpha-N-acetyl-neuraminide alpha-2,8-sialyltransferase 3	1.82
NM_001042550	SMC2	structural maintenance of chromosomes 2	1.82
NM_004703	*RABEP1	rabaptin, RAB GTPase binding effector protein 1	1.82
NM_001127370	CDCA7L	cell division cycle associated 7-like	1.81
NM_006982	ALX1	ALX homeobox 1	1.81
NM_018728	*MYO5C	myosin VC	1.81
NR_003719	NBPF22P	neuroblastoma breakpoint family, member 22 (pseudogene)	1.81
NM_012404	ANP32D	acidic (leucine-rich) nuclear phosphoprotein 32 family, member D	1.81
NM_001004701	OR4C16	olfactory receptor, family 4, subfamily C, member 16	1.80
NM_018640	LMO3	LIM domain only 3 (rhombotin-like 2)	1.80
NM_000175	GPI	glucose-6-phosphate isomerase	1.78
NM_032581	*FAM126A	family with sequence similarity 126, member A	1.78
NM_017918	CCDC109B	coiled-coil domain containing 109B	1.78
NM_001083926	ASRGL1	asparaginase like 1	1.77
NM_019120	*PCDHB8	protocadherin beta 8	1.76
NM_017697	ESRP1	epithelial splicing regulatory protein 1	1.76
NM_012214	MGAT4A	mannosyl (alpha-1,3-)-glycoprotein beta-1,4-N-acetylglucosaminyltransferase, isozyme A	1.75
NM_018492	PBK	PDZ binding kinase	1.75
NM_001114120	DEPDC1	DEP domain containing 1	1.75
NM_001040284	PAPD5	PAP associated domain containing 5	1.73
NM_015214	DDHD2	DDHD domain containing 2	1.73
NM_016060	MED31	mediator complex subunit 31	1.73
NM_007246	KLHL2	kelch-like 2, Mayven (Drosophila)	1.72
NM_182633	ZNF713	zinc finger protein 713	1.72
NM_001262	CDKN2C	cyclin-dependent kinase inhibitor 2C	1.71
NM_015986	*CRLF3	cytokine receptor-like factor 3	1.70
AF090937	SORBS2	sorbin and SH3 domain containing 2	1.70
NM_007109	*TCF19	transcription factor 19	1.70
NM_175768	GRIK2	glutamate receptor, ionotropic, kainate 2	1.69
NM_001004475	OR10T2	olfactory receptor, family 10, subfamily T, member 2	1.69
NM_015146	SEPT8	septin 8	1.69
NM_024775	GEMIN6	gem (nuclear organelle) associated protein 6	1.69
NM_004853	STX8	syntaxin 8	1.68
NM_138966	NETO1	neuropilin (NRP) and tolloid (TLL)-like 1	1.68
NM_033512	TSPYL5	TSPY-like 5	1.68
NM_002004	FDPS	farnesyl diphosphate synthase	1.68

NM_001113402	AMN1	antagonist of mitotic exit network 1 homolog	1.67
NR_026550	BRP44	brain protein 44	1.67
NM_001145065	FAM190A	family with sequence similarity 190, member A	1.66
NM_013250	ZNF215	zinc finger protein 215	1.66
NM_023936	*MRPS34	mitochondrial ribosomal protein S34	1.66
NM_001100816	MED7	mediator complex subunit 7	1.66
NM_001493	GDI1	GDP dissociation inhibitor 1	1.65
NM_005507	*CFL1	cofilin 1 (non-muscle)	1.64
NM_003757	EIF3I	eukaryotic translation initiation factor 3, subunit I	1.64
NM_001221	*CAMK2D	calcium/calmodulin-dependent protein kinase II delta	1.64
NM_032117	MND1	meiotic nuclear divisions 1 homolog (S. cerevisiae)	1.64
NM_003545	HIST1H4E	histone cluster 1, H4e	1.64
NM_030806	C1orf21	chromosome 1 open reading frame 21	1.64
NM_001967	EIF4A2	eukaryotic translation initiation factor 4A2	1.63
NM_005669	REEP5	receptor accessory protein 5	1.62
NM_020236	MRPL1	mitochondrial ribosomal protein L1	1.62
NM_005649	ZNF354A	zinc finger protein 354A	1.62
NM_002803	PSMC2	proteasome 26S subunit, ATPase, 2	1.61
NM_000732	CD3D	CD3d molecule, delta (CD3-TCR complex)	1.61
NM_015252	EHBP1	EH domain binding protein 1	1.61
NM_002532	NUP88	nucleoporin 88kDa	1.61
NR_027779	*TTLL1	tubulin tyrosine ligase-like family, member 1	1.59
NM_001991	EZH1	enhancer of zeste homolog 1 (Drosophila)	1.59
NM_005319	HIST1H1C	histone cluster 1, H1c	1.59
NM_015194	MYO1D	myosin ID	1.59
NM_152622	MIER3	mesoderm induction early response 1, family member 3	1.59
NM_006597	HSPA8	heat shock 70kDa protein 8	1.59
NM_001613	ACTA2	actin, alpha 2, smooth muscle, aorta	1.59
NM_152775	CCDC110	coiled-coil domain containing 110	1.58
NM_017665	ZCCHC10	zinc finger, CCHC domain containing 10	1.58
NM_006186	*NR4A2	nuclear receptor subfamily 4, group A, member 2	1.57
XR_042118	LOC388882	hypothetical LOC388882	1.57
NM_207445	C15orf54	chromosome 15 open reading frame 54	1.57
NM_019060	CRCT1	cysteine-rich C-terminal 1	1.56
NM_198439	*KBTBD3	kelch repeat and BTB (POZ) domain containing 3	1.56
NM_001112724	*STK32A	serine/threonine kinase 32A	1.56
NM_152515	CKAP2L	cytoskeleton associated protein 2-like	1.56
NM_005184	CALM3	calmodulin 3 (phosphorylase kinase, delta)	1.55
NM_001079519	FAM177A1	family with sequence similarity 177, member A1	1.55
NM_004453	ETFDH	electron-transferring-flavoprotein dehydrogenase	1.54
NM_013233	STK39	serine threonine kinase 39	1.54
NM_012383	OSTF1	osteoclast stimulating factor 1	1.54
NM_014279	OLFM1	olfactomedin 1	1.53

NM_194463	RNF128	ring finger protein 128	1.53
NM_016048	*ISOC1	isochorismatase domain containing 1	1.52
NM_004331	*BNIP3L	BCL2/adenovirus E1B 19kDa interacting protein 3-like	1.52
NM_032785	AGBL4	ATP/GTP binding protein-like 4	1.51
NM_015421	TMEM186	transmembrane protein 186	1.51
NM_000436	OXCT1	3-oxoacid CoA transferase 1	1.51
NM_003558	PIP5K1B	phosphatidylinositol-4-phosphate 5-kinase, type I, beta	1.51
NM_138578	BCL2L1	BCL2-like 1	1.51
NM_024528	NKAP	NFKB activating protein	1.50
AY422473	*C9orf93	chromosome 9 open reading frame 93	1.50
NM_013339	ALG6	asparagine-linked glycosylation 6, alpha-1,3-glucosyltransfase	1.50
NM_181684	KRTAP12-2	keratin associated protein 12-2	1.50
NM_001135155	*DPF1	D4, zinc and double PHD fingers family 1	-1.51
NM_007225	*NXPH3	neurexophilin 3	-1.51
NM_212460	ARL4A	ADP-ribosylation factor-like 4A	-1.51
NM_022036	GPRC5C	G protein-coupled receptor, family C, group 5, member C	-1.51
NM_018654	GPRC5D	G protein-coupled receptor, family C, group 5, member D	-1.51
NM_006570	RRAGA	Ras-related GTP binding A	-1.52
NM_052923	*SCAND3	SCAN domain containing 3	-1.53
NM_002968	*SALL1	sal-like 1 (Drosophila)	-1.53
NM_006541	GLRX3	glutaredoxin 3	-1.54
NM_002550	OR3A1	olfactory receptor, family 3, subfamily A, member 1	-1.54
NM_003598	TEAD2	TEA domain family member 2	-1.55
NM_199355	*ADAMTS18	ADAM metallopeptidase with thrombospondin type 1 motif, 18	-1.55
NM_031484	MARVELD1	MARVEL domain containing 1	-1.56
NM_033446	*FAM125B	family with sequence similarity 125, member B	-1.56
NM_002047	GARS	glycyl-tRNA synthetase	-1.56
NM_032935	MT4	metallothionein 4	-1.57
NM_030965	ST6GALNAC5	ST6 (alpha-N-acetyl-neuraminyl-2,3-beta- galactosyl-1,3)-N-acetylgalactosaminide alpha-2,6-sialyltransferase 5	-1.57
NM_004335	BST2	bone marrow stromal cell antigen 2	-1.57
NM_000146	FTL	ferritin, light polypeptide	-1.58
NM_012324	MAPK8IP2	mitogen-activated protein kinase 8 interacting protein	-1.58
NM_004911	*PDIA4	protein disulfide isomerase family A, member 4	-1.59
NM_003979	GPRC5A	G protein-coupled receptor, family C, group 5, member A	-1.59
NM_017763	RNF43	ring finger protein 43	-1.61
NM_015564	LRRTM2	leucine rich repeat transmembrane neuronal 2	-1.61
NM_138704	NDNL2	necdin-like 2	-1.62

NM_175874	C12orf60	chromosome 12 open reading frame 60	-1.62
NM_003507	FZD7	frizzled homolog 7 (Drosophila)	-1.62
NM_000317	PTS	6-pyruvoyltetrahydropterin synthase	-1.62
NM_178536	LCN12	lipocalin 12	-1.63
NM_001006605	FAM69A	family with sequence similarity 69, member A	-1.63
NM_002615	SERPINF1	serpin peptidase inhibitor, clade F (alpha-2 antiplasmin, pigment epithelium derived factor), member 1	-1.63
NM_000197	HSD17B3	hydroxysteroid (17-beta) dehydrogenase 3	-1.63
NM_006347	PPIH	peptidylprolyl isomerase H (cyclophilin H)	-1.63
NM_005841	SPRY1	sprouty homolog 1, antagonist of FGF signaling	-1.64
NM_002124	HLA-DRB1	major histocompatibility complex, class II, DR beta 1	-1.64
NM_003395	WNT9A	wingless-type MMTV integration site family, member 9A	-1.65
NM_032208	ANTXR1	anthrax toxin receptor 1	-1.65
NM_032873	UBASH3B	ubiquitin associated and SH3 domain containing B	-1.65
NM_014670	BZW1	basic leucine zipper and W2 domains 1	-1.65
NM_003615	SLC4A7	solute carrier family 4, sodium bicarbonate cotransporter, member 7	-1.66
NM_006933	SLC5A3	solute carrier family 5 (sodium/myo-inositol cotransporter), member 3	-1.68
NM_001113567	*C17orf76	chromosome 17 open reading frame 76	-1.68
NM_000510	*FSHB	follicle stimulating hormone, beta polypeptide	-1.69
NM_004479	FUT7	fucosyltransferase 7 (alpha (1,3) fucosyltransferase)	-1.73
NM_002144	*HOXB1	homeobox B1	-1.73
NM_003770	KRT37	keratin 37	-1.73
NM_078487	*CDKN2B	cyclin-dependent kinase inhibitor 2B	-1.73
NM_024787	*RNF122	ring finger protein 122	-1.73
NM_001042481	FRMD6	FERM domain containing 6	-1.79
NM_023917	TAS2R9	taste receptor, type 2, member 9	-1.86
NM_001839	CNN3	calponin 3, acidic	-1.90
NM_001904	CTNNB1	catenin (cadherin-associated protein), beta 1, 88kDa	-1.94
NM_016433	*GLTP	glycolipid transfer protein	-1.95
NM_001004487	OR13J1	olfactory receptor, family 13, subfamily J, member 1	-1.95
NM_033184	*KRTAP2-4	keratin associated protein 2-4	-1.99
NM_207032	EDN3	endothelin 3	-2.03
NM_020640	*DCUN1D1	DCN1, defective in cullin neddylation 1, domain containing 1	-2.04
NM_006189	OMP	olfactory marker protein	-2.10
NM_002010	FGF9	fibroblast growth factor 9 (glia-activating factor)	-2.14
NM_181619	KRTAP21-1	keratin associated protein 21-1	-2.16
NM_019590	KIAA1217	KIAA1217	-2.39
NM_181611	*KRTAP19-5	keratin associated protein 19-5	-2.86
NR_024873	MCART1	mitochondrial carrier triple repeat 1	-3.21
NM_000762	CYP2A6	cytochrome P450, family 2, subfamily A, polypeptide 6	-3.32
